# MCP-1 is increased in patients with CFS and FM, whilst several other immune markers are significantly lower than healthy controls

**DOI:** 10.1016/j.bbih.2020.100067

**Published:** 2020-03-28

**Authors:** Nina Groven, Egil Andreas Fors, Astrid Kamilla Stunes, Solveig Klæbo Reitan

**Affiliations:** a Department of Mental Health, Faculty of Medicine and Health Sciences, Norwegian University of Science and Technology (NTNU), Trondheim, Norway; b Department of Psychiatry and Clinical Neurophysiology, St. Olav’s University Hospital, Trondheim, Norway; c Department of Public Health and Nursing, Faculty of Medicine and Health Sciences, Norwegian University of Science and Technology (NTNU), Trondheim, Norway; d Department of Clinical and Molecular Medicine, Faculty of Medicine and Health Sciences, Norwegian University of Science and Technology (NTNU), Trondheim, Norway; e Medical Clinic, St. Olav’s University Hospital, Trondheim, Norway

**Keywords:** Fibromyalgia, FM, Chronic fatigue syndrome, CFS, MCP-1, Cytokines, Chemokines, Inflammation

## Abstract

The role of the immune system in the pathogenesis of Fibromyalgia (FM) and Chronic fatigue syndrome (CFS) is not clear. We have previously reported increased levels of C-reactive protein (CRP) in these patient groups compared to healthy controls and wanted to further explore the levels of circulating immune markers in these populations.

The population consisted of three groups, 58 patients with FM, 49 with CFS and 54 healthy controls. All participants were females aged 18–60. Patients were recruited from a specialised university hospital clinic and controls were recruited by advertisement among the staff and students at the hospital and university. Plasma levels of Interferon (IFN)-γ, Interleukin (IL)-1β, IL-1ra, IL-4, IL-6, IL-8, IL-10, IL-17, Interferon gamma-induced protein (IP)-10, Monocyte Chemoattractant Protein (MCP)-1, Transforming Growth Factor (TGF)-β1, TGF-β2, TGF-β3 and Tumour Necrosis Factor (TNF)-α were analysed by multiplex. Differences between the three groups CFS, FM and controls, were analysed by Kruskal Wallis tests.

MCP-1 was significantly increased in both patient groups compared to healthy controls. IL-1β, Il-4, IL-6, TNF-α, TGF-β1, TGF-β2, TGF-β3, IL-10 and IL17 all were significantly lower in the patient groups than healthy controls. IFN-γ was significantly lower in the FM group. For IL-8, IL-10 and IL-1ra there were no significant difference when controlled for multiple testing.

In conclusion, in our material MCP-1 seems to be increased in patients both with CFS and with FM, while several other immune markers are significantly lower in patients than controls.

## Highlights

• MCP-1 is significantly increased in CFS and Fibromyalgia compared to controls.
• CFS and Fibromyalgia share common features of inflammation.
• Decreased IL-1β, IL-4, IL-6, TNF-α, TGF-β1, TGF-β2, TGF-β3, IL-10 and IL-17A are found in both CFS and Fibromyalgia.

## 1 Introduction

Chronic fatigue syndrome (CFS) and fibromyalgia (FM) are challenging conditions affecting 0.5–8% of the population, with both socioeconomic and personal burdens. Aetiologies of these conditions are not well understood (Singh et al., 2019; Yang et al., 2019b). The two syndromes are classified distinctly in ICD-10 and ICD-11. Diagnostic criteria for CFS in the majority of previous research is based on the 1994 Fukuda Criteria (Fukuda et al., 1994) and diagnosis of FM has until recently been based on the 1990 ACR criteria (Wolfe et al., 1990). Fatigue and widespread pain are common symptoms for most CFS and FM patients, where the emphasis on one of the symptoms, fatigue or pain, has been typical differentiating characteristic for CFS or FM, respectively, even though this paradigm has changed somewhat with the new SEID criteria (Institute of Medicine [IOM], 2015) for CFS and 2016 Fibromyalgia criteria (Wolfe et al., 2016).

Involvement of immunological mechanisms have been postulated in the pathology of CFS and FM patients (Coskun Benlidayi, 2019; Morris et al., 2019). The symptoms of CFS and FM mimic those seen in different inflammatory disorders (Jonsjo et al., 2020). Inflammation is related to cytokines and chemokines (immune markers) regulating the immune response. However, cytokines and chemokines can also influence behaviour and mental state (*e.g.* by inducing fatigue, depression, and hyperalgesia) (Hestad et al., 2009). Thus, comparing levels of immune markers in patients may indicate immune activity and even reveal pathological mechanisms in patients.

Although a few studies (Iacob et al., 2016; Light et al., 2012; Nakamura et al., 2010; Scully et al., 2010) have compared immunological markers between CFS and FM, most studies focus on each disorder separately, and do not consider the heterogenicity in and overlap between the two conditions (Backryd et al., 2017; Yang et al., 2019b). Importantly, studies including levels of several immunological factors in larger samples are lacking.

We previously have reported a tendency (*p* ​= ​.056) towards increased plasma levels of TNF-α in a group of 20 CFS patients compared to 20 healthy controls (Groven et al., 2018). Furthermore, we have reported increased hsCRP in both FM and CFS in a larger group of patients compared to controls (Groven et al., 2019). Based on these findings, we therefore hypothesized a pro-inflammatory pattern in CFS and FM patients with elevated pro-inflammatory immune markers (like IFN-γ, IL-1β, IL-6, IL-8, Interferon gamma-induced protein (IP)-10, TNF-α, and MCP-1); lower levels of anti-inflammatory (like IL-1ra and IL-10), and regulatory (like IL-4, TGF-β1, TGF-β2, TGF-β3, and IL-17) immune markers.

## 2 Method

### 2.1 Sample population

#### 2.1.1 Patient groups

As previously reported (Groven et al., 2019), patients were female, non-pregnant patients aged 18–60 years admitted to the Multidisciplinary Pain Centre at St. Olav’s University Hospital, Norway. Patients were referred to this centre by general practitioners in Mid-Norway.

Each participant went through a comprehensive clinical examination and was thoroughly evaluated by an expert team of medical doctors, physiotherapists and psychologists. All patients were assessed by using the 1990 ACR (Wolfe et al., 1990) and the 1994 Fukuda criteria (Fukuda et al., 1994) as both were still used as diagnostic tools in the clinic during the recruitment period. FM patients (*n* ​= ​58) were eligible if they fulfilled the 1990 ACR criteria (Wolfe et al., 1990). CFS patients (*n* ​= ​49) were eligible if they fulfilled the Fukuda diagnostic criteria (Fukuda et al., 1994). Exclusion criteria were in accordance with diagnostic criteria including known inflammatory diseases.

#### 2.1.2 Healthy controls

A group of 53 healthy females aged 18–60 years was consecutively recruited by advertising through websites among the staff of the Norwegian University of Science and Technology (NTNU) and St. Olav’s University Hospital. Their health was assessed by conducting a structured medical history and by using questionnaires included in this study measuring the symptoms of CFS and FM (see 2.4 Questionnaires and 2.5 Interview).

### 2.2 Procedure, study design and ethics

The CFS patients were informed about the study by a letter sent by the hospital prior to or shortly after their clinical evaluation or given during their evaluation at the centre. The FM patients were given an information letter by the staff during the clinical examination. Both patient groups were then contacted by phone and asked for participation in this study by a member of staff, and an appointment was scheduled for those who accepted to join the study.

The study assessment lasted approximately 30–40 ​min and included an interview, questionnaires, and blood sampling. All data were collected by NG in the period from March 2015 to December 2016. The order of the assessments was random.

The study was approved by the Regional Committee for Medical and Health Research Ethics (REK 2014/711). Written informed consent was obtained from all participants.

### 2.3 Questionnaires

#### 2.3.1 Hospital Anxiety and Depression Scale

The Hospital Anxiety and Depression Scale (HADS) is a validated, self-complete scale (Bjelland et al., 2002; Zigmond and Snaith, 1983) for monitoring depressive and anxiety symptoms. It is summarised as HADS-D (depression) and HADS-A (anxiety). The potential HADS sub-scores range from 0 to 21 with high scores being suggestive of more symptoms.

#### 2.3.2 Chalder fatigue scale

The Chalder Fatigue Scale (Chalder et al., 1993; Loge et al., 1998) is used to evaluate (the severity of) fatigue in CFS patients. The total sum of each of the 11 items, scored on a 0–3 Likert scale, total score ranging from 0 to 33, is applied; higher scores imply more severe fatigue.

#### 2.3.3 Pain – Numeric Rating Scale

A Numeric Rating Scale (NRS) was used to evaluate the subjective feeling of experienced pain on average (for the last week and is taken from the Brief Pain Inventory (Cleeland, 1991; Klepstad et al., 2002) which is a Likert scale ranging from 0 (“no pain”) to 10 (“maximal possible pain”).

#### 2.3.4 Fibromyalgia Survey Diagnostic Criteria

Fibromyalgia Survey Diagnostic Criteria (FSDC) was used in this study for quality assessment of the ACR 1990 criteria used for inclusion. The FSDC is based on the Fibromyalgia Survey Questionnaire developed in 2010/2011 (Wolfe et al., 2010, 2011) and later revised in 2016 (Wolfe et al., 2016). FSDC is a self-report questionnaire used for diagnostics and classification in epidemiological studies. The FSDC consists of two sub-scales: Widespread Pain Index, scores 0–9; and Symptom Severity Scale, scores 0–12. Widespread Pain Index and Symptom Severity Scale are summarised into a third, score, *i.e.* the Fibromyalgia Severity (FS) score, ranging from 0 *(no symptoms*) to 31 (*most severe symptoms*) and indicate the severity of symptoms.

### 2.4 Interview and anthropometrics

For each participant, age, height, and weight as well as a structured clinical interview were recorded. History regarding infections, immune disorders, illness in general (somatic as well as psychiatric), medication, menstrual cycle, use of contraceptives, status of menopause, duration of illness (if applicable), and level of physical activity during the previous two weeks were recorded. The latter was scored on a scale from 1 (bedridden) to 4 (conducting regular exercise more than two times per week).

### 2.5 Blood sampling and analyses

There were no restrictions, such as fasting, dietary restrictions or use of medication, given prior to blood sampling. Samples were analysed at the clinical laboratory at St. Olav’s Hospital, Trondheim. Samples were screened for deviating levels of white blood cells (WBC), CRP, and serology against mycoplasma pneumonia, borrelia burgdorferi, cytomegalo-, Epstein-Barr -, hepatitis B - and hepatitis C virus. Any sign of infection led to exclusion from the study.

Blood samples for immune markers Interferon (IFN)-γ, Interleukin (IL)-1β, IL-1ra, IL-4, IL-6, IL-8, IL-10, IL-17, Interferon gamma-induced protein (IP)-10, Monocyte Chemoattractant Protein (MCP)-1, Transforming Growth Factor (TGF)-β1, TGF-β2, TGF-β3 and Tumor Necrosis Factor (TNF)-α were collected in EDTA plasma tubes, immediately put on ice, centrifuged (1500g, 15 ​min, 4 ​°C), aliquoted into cryovials and frozen at −80 ​°C until further analyses.

Immune markers were analysed using multianalyte profiling Milliplex MAP assay (Millipore, Billerica, MA) with a Bio-plex 2000 and the Bio-plex manager software (Biorad, Hercules, CA). The percentages of samples that were below the detection limits ranged from 0 to 29%. All samples analysed had levels of IP-10, MCP-1, TNF-α, TGF-β1 and TGF-β2 above the manufacturer’s detection limits (8.6 ​pg/mL, 1.9 ​pg/mL, 0.7 ​pg/mL, 3.9 ​pg/mL and 1.9 ​pg/mL, respectively). Detection limits for IFN-γ, IL-1β, IL-1ra, IL-4, IL-6, IL-8, TGF-β3, IL-10 and IL-17A were based on the lowest detected concentrations in our samples: 0.43 ​pg/mL, 0.05 ​pg/mL, 1.6 ​pg/mL, 0.31 ​pg/mL, 0.40 ​pg/mL, 0.05 ​pg/mL, 0.91 ​pg/mL, 1.15 ​pg/mL, 6.36 ​pg/mL, respectively. Samples below the detection limits for these nine cytokines were set to half of the detection limits (0.215 ​pg/mL, 0.025 ​pg/mL, 0.8 ​pg/mL, 0.155 ​pg/mL, 0.20 ​pg/mL, 0.025 ​pg/mL, 0.46 ​pg/mL, 0.58 ​pg/mL and 3.18 ​pg/mL, respectively). All analyses were performed according to the manufacturer’s protocol.

### 2.6 Statistical analysis

The statistical analyses were performed using the Statistical SoftWare Package (SPSS) Statistics for Windows, version 22. All variables were tested for normality and homogeneity by using the Shapiro-Wilk tests and visual inspection of histograms and Q-Q-plots.

The data consisted of a considerable number of samples below the detection limit. Transformation of the data did not improve this bias, and the Kruskal-Wallis ranks test was applied for comparison between groups. Dunn’s test was used for post-hoc analysis of pair-wise group comparisons. Associations between variables were analysed by Spearman’s Rank-Order Correlation analyses (ρ). Confounding factors were defined as variables with significant associations of *p* ​< ​.05. A conservative approach was taken to account for multiple comparisons between groups, and these results were considered significant at *p* ​< ​.01. Results reaching levels of *p* ​< ​.05 were added for comparison purposes.

## 3 Results

### 3.1 Population

A total of 160 participants were included in this study, consisting of 49 CFS patients, 58 FM patients, and 53 healthy controls. The CFS patients in this study were younger than the FM and control group (*p* ​< ​.001 and *p* ​= ​.010, respectively). The FM group had higher BMI than the CFS and control groups (*p* ​= ​.007 and *p* ​= ​.049, respectively). Both patient groups had significantly higher HADS depression and anxiety scores, fatigue scores, pain scores (NRS) and FS scores compared to controls. The demographic and clinical characteristics of the sample population is summarised in Table 1.

**Table 1:** Descriptive of age, Body Mass Index (BMI), depression, anxiety, fatigue - and fibromyalgia scores in CFS, FM and controls.

Parameter	CFS				FM				Control				*p*^a^
	(*n* ​= ​49)				(*n* ​= ​58)				(*n* ​= ​53)				
	Missing	*M (SD)*	*Mdn*	*Range*	Missing	*M (SD)*	*Mdn*	*Range*	Missing	*M (SD)*	*Mdn*	*Range*	
	(*n*)			*(min– max)*	(*n*)			*(min– max)*	(*n*)			*(min– max)*	
Age	0	33.8 (11.3)	35.0	18–60	0	42.0 (9.1)	42.5	22–60	0	39.4 (10.4)	39.0	23–59	<.001
BMI	2	24.0 (3.6)	23.1	18.1–34.6	1	26.7 (5.6)	25.7	16.3–40.4	0	24.7 (4.0)	23.8	16.3–41.7	.017
HADS depression	0	6.0 (4.2)	5.0	0–17	1	6.4 (3.9)	6.0	0–16	1	1.3 (1.8)	1.0	0–8	<.001
HADS anxiety	0	5.9 (4.6)	5.0	0–19	1	8.4 (4.1)	8.0	0–17	1	3.2 (2.6)	3.0	0–10	<.001
Fatigue score	0	36.5 (5.3)	37.0	23–44	1	33.5 (5.3)	34.0	18–44	1	21.3 (3.2)	21.0	14–33	<.001
Pain NRS	0	3.9 (2.0)	4.0	0–9	0	6.0 (1.8)	6.0	0–10	2	0.9 (1.2)	1.0	0–5	<.001
FS score	0	15.2 (5.5)	13.0	7–29	1	20.1 (5.2)	20.0	3–30	1	3.1 (2.5)	3.0	0–11	<.001

Note: BMI ​= ​body mass index. HADS = Hospital Anxiety and Depression Scale. FS = Fibromyalgia Severity.

a Kruskall-Wallis test.

### 3.2 Immune markers

Comparisons of cytokine levels between the three groups are shown in Table 2. The Kruskal-Wallis test showed significant group differences for 12 of the 14 cytokines: INF-γ, IL-1ra, IL-1β, IL-4, IL-6, MCP-1, TNF-α, TGF-β1, TGF-β2, TGF-β3, IL-10 and IL-17A. Two cytokines did not show any significant group differences, *i.e*. IL-8 and IP-10.

**Table 2:** Plasma levels of cytokines (pg/mL) in CFS, FM and healthy controls.

Parameter	CFS			FM			Control			*p*^b^
	(*n* ​= ​48)			(*n* ​= ​58)			(*n* ​= ​53)			
	*M (SD)*	*Mdn*	*IQR*^a^	*M (SD)*	*Mdn*	*IQR*^a^	*M (SD)*	*Mdn*	*IQR*	
INF-γ^c^	175.01 (879.68)	29.51	11.58–48.67	35.33 (90.19)	17.59	10.44–26.39	92.00 (318.18)	30.31	17.13–54.77	<.001∗
IL-1ra	226.92 (1052.08)	20.40	0.80–125.46	113.74 (255.37)	25.39	0.80–83.88	135.73 (243.92)	61.10	11.30–123.75	.031
IL-1β^d^	28.08 (143.74)	4.23	0.56–6.68	3.86 (3.68)	2.75	0.93–6.16	10.53 (16.26)	7.07	4.25–11.66	<.001∗
IL-4^d^	43.07 (219.89)	5.31	0.16–19.26	24.66 (96.43)	1.57	0.16–12.05	47.72 (48.56)	34.51	21.16–61.90	<.001∗
IL-6^d^	13.69 (49.4)	2.28	0.20–7.05	5.22 (6.27)	3.45	0.20–7.14	13.35 (23.17)	8.04	4.90–15.16	<.001∗
IL-8^e^	16.75 (61.55)	1.74	0.20–11.20	10.49 (23.27)	1.55	0.03–9.17	14.27 (28.33)	3.41	1.00–12.62	.205
IP-10^e^	382.69 (204.40)	334.80	275.63–438.63	381.84 (152.68)	326.63	284.48–443.32	374.76 (191.74)	310.43	264.84–437.66	.750
MCP-1^f^	221.13 (62.08)	210.78	187.83–241.11	209.97 (71.69)	202.62	169.89–235.42	190.17 (75.71)	183.25	160.36–198.26	<.001∗
TNF-α^d^	37.28 (155.64)	14.81	10.20–19.32	13.37 (5.24)	12.66	9.79–17.47	22.95 (26.03)	18.59	13.87–24.95	<.001∗
TGF-β1^d^	3806.11 (3007.46)	2783.11	1476.36–5632.41	3334.18 (2174.34)	2400.00	1491.95–5166.31	5436.44 (1899.64)	5650.65	4261.62–6675.55	<.001∗
TGF-β2^d^	390.72 (156.38)	414.70	252.69–531.69	404.48 (146.21)	341.07	267.33–539.59	559.94 (116.38)	545.91	499.86–611.57	<.001∗
TGF-β^d^	32.20 (32.68)	18.42	4.40–59.80	30.91 (27.85)	17.70	7.48–57.10	57.90 (30.46)	55.78	39.02–74.06	<.001∗
IL-10^c^	19.50 (27.75)	15.32	7.28–22.98	14.50 (9.03)	13.56	6.85–20.08	19.20 (6.14)	18.56	15.65–22.55	.003∗
IL-17A^c^	114.58 (92.66)	115.56	60.53–142.48	98.51 (43.06)	104.66	66.44–130.38	130.61 (40.12)	128.44	109.21–145.34	.002∗

Note: *IQR* ​= ​inter-quartile range. ∗Significance *p* ​< ​.01.

a Values below the detection limit were set to half detection limit.

b Kruskall-Wallis test.

c FM has significant lower value compared to both CFS and controls.

d Both FM and CFS have significantly lower values than controls.

e No significant differences between any groups.

f Both FM and CFS have significantly higher values than controls.

Both patient groups had significantly lower plasma levels than controls for the following seven cytokines: IL-1β, IL-4, IL-6, TNF-α, TGF-β1, TGF-β2, and TGF-β3. CFS and FM patients could not be distinguished between each other for these cytokines. Post-hoc Dunn’s tests showed that for INF-γ, FM patients had significantly lower ranks than CFS patients and controls (*p* ​< ​.001), and CFS patients and controls could not be distinguished from each other (*p* ​< ​.05). FM patients also had significantly lower plasma levels compared to controls for IL-10 and IL-17A (*p* ​< ​.001), but the FM group was not different from CFS patients (*p* ​= ​.220; and *p* ​= ​.339, respectively) and CFS patients did not show any differences compared to controls (*p* ​< ​.05).

For MCP-1, both CFS and FM patients had significantly higher levels than the control group (*p* ​< ​.001). However, the patient groups could not be distinguished from each other (*p* ​= ​.235).

### 3.3 Confounding factors

#### 3.3.1 Age

Age ranged from 18 to 60 years for the total study population. In the total study population, age was positively associated to levels of IP-10 and MCP-1 (*p* ​= ​.002, ρ ​= ​0.249; and *p* ​< ​.001, ρ ​= ​0.301).^1^

#### 3.3.2 BMI

BMI was positively correlated with IL-6 (*p* ​= ​.021, ρ ​= ​0.158), IL-8 (*p* ​= ​.039, ρ ​= ​0.165), and MCP-1 (*p* ​= ​.055, ρ ​= ​0.154) in the total population.^1^

Age and BMI were not associated with other immune markers for the total population sample.

#### 3.3.3 Questionnaires

##### 3.3.3.1 Anxiety and depression

For the whole study population, the HADS depression score showed a positive correlation with IP-10 and MCP-1 (*p* ​= ​.008, ρ ​= ​0.211; and *p* ​= ​.008, ρ ​= ​0.212), and negative correlation with IL-1β, IL-4, IL-6, TGF-β1, TGF-β2, TGF-β3, and TNF-α (*p* ​= ​.010, ρ ​= ​−0.204; *p* ​< ​.001, ρ ​= ​−0.252; *p* ​= ​.010, ρ ​= ​−0.206; *p* ​= ​.001, ρ ​= ​−0.264; *p* ​< ​.001, ρ ​= ​−0.295; *p* ​= ​.001, ρ ​= ​−0.264; and *p* ​< ​.012, ρ ​= ​−0.199 respectively). For the total study population, HADS anxiety scores showed a negative correlation with IL-4 and IL-6 (*p* ​= ​.021, ρ ​= ​−0.184; and *p* ​= ​.023, ρ ​= ​−0.182). When examining the groups of participants separately, only a positive correlation between HADS depression and IP-10 in the CFS group remained (*p* ​= ​.048, ρ ​= ​0.287).^1^

##### 3.3.3.2 Chalder fatigue scale

For the whole study population, levels of fatigue did not correlate with any of the immune markers. When studying the groups separately, the only significant finding was a negative correlation between fatigue levels and MCP-1 in the FM group (*p* ​= ​.029, ρ ​= ​−0.290).^1^

##### 3.3.3.3 Pain NRS

There were negative associations between the subjective feeling of the average experienced pain for the past week (NRS) and, in addition to IL-8, the same immune markers that also were lower in both patient groups compared to controls (INF-γ, IL-1ra, IL-1β, IL-4, IL-6, TNF-α, TGF-β1, TGF-β2, TGF-β3, IL-10 and IL-17A; see section 3.), and a positive correlation between this NRS score and MCP-1. There were no associations between the subjective feeling of the average experienced pain for the past week (NRS) and any of the immune markers measured when analysing CFS, FM and controls separately.^1^

##### 3.3.3.4 Fibromyalgia: ACR 1990 and FSDC

In the CFS group, 13 (26.5%) patients fulfilled the ACR 1990 criteria (Wolfe et al., 1990) while 21 (38.8%) patients fulfilled the new 2016 FSDC criteria (Wolfe et al., 2016). For the FM group, all patients fulfilled the ACR 1990 criteria by inclusion. Forty-nine (84.5%) of the FM patients also fulfilled the FSDC 2016 criteria. The sensitivity and specificity of how well the ACR 1990 criteria predicted the new FSDC 2016 criteria were both 0.88 in our population sample.

There were negative associations between the FS score and the same immune markers that also were lower in both patient groups compared to controls (INF-γ, IL-1ra, IL-1β, IL-4, IL-6, TNF-α, TGF-β1, TGF-β2, TGF-β3, IL-10 and IL-17A; see section 3.2), and a positive correlation between the FS score MCP-1.^1^

#### 3.3.4 Interview

##### 3.3.4.1 Menopause

Differences in IP-10 (*p* ​= ​.001, *U* ​= ​487.0) and MCP-1 (*p* ​< ​.001, *U* ​= ​379.0) were found before versus after menopause, with higher levels of both cytokines after menopause. Only 4 in the CFS group, 7 in the FM group and 5 control participants reported having reached menopause. No associations were seen for other cytokines.

##### 3.3.4.2 Duration of illness

The time that had elapsed from onset of illness to the day of the interview was recorded in years. Patients were divided into those reporting having their illness lasting less than three years (short duration) (22%), and the those reporting having their illness lasting longer than three years (long duration) (78%). Shorter duration of illness for CFS patients had significantly lower levels compared to CFS patients with longer duration of illness for TNF-α, TGF-β1, TGF-β2, TGF-β3 and IL-10 (*p* ​≤ ​.001, for these cytokines). Comparing CFS patients with longer duration of illness to the control group, showed that CFS patients had lower levels compared to controls for TGF-β1, TGF-β2, and TGF-β3 (*p* ​= ​.005, *p* ​< ​.001, and *p* ​= ​.004, respectively), but these patients could no longer be distinguished from controls on TNF-α and IL-10 (*p* ​= ​.073 and *p* ​= ​.673, respectively).

There were no differences between FM patients with short or long duration of illness for any immune markers measured in this study.

##### 3.3.4.3 Activity level

A negative association was found between higher levels of activity and MCP-1 (*p* ​= ​.005, ρ ​= ​−0.223) for the total study population.^1^

##### 3.3.4.4 Other

No associations were found between immune markers and smoking, type and use of medication, use of birth control and stage of menstrual cycle.

## 4 Discussion

The two patient groups (CFS and FM) had significantly lower circulating levels compared to healthy controls for the following nine cytokines: IL-1β, IL-4, IL-6, TNF-α, TGF-β1, TGF-β2, TGF-β3, IL-10 and IL-17A, and the two patient groups could not be distinguished from each other. INF-γ was significantly lower in the FM group compared to both CFS and controls. No significant group differences were observed for the circulating levels of IL-8 and IP-10.

### 4.1 MCP-1

In the current study of several plasma immune markers, only pro-inflammatory MCP-1 was significantly increased in both patient groups, CFS and FM, compared to healthy controls.

MCP-1 (CCL2) is a potent pro-inflammatory chemokine increasing inflammation by directing migration and infiltration of monocytes/macrophages to the site of activity (Deshmane et al., 2009). Though MCP-1 is central in inflammation, including neuroinflammation (Conductier et al., 2010) there are few studies on the role of circulating MCP-1 in FM and CFS.

The increased MCP-1 levels for FM patients in our study is in accordance with a study by Zhang et al. (2008) where plasma MCP-1 levels were increased in 92 FM patients compared to 48 healthy controls. Similarly, *ex vivo* MCP-1 release by blood monocytes from 25 FM patients was increased compared to release from monocytes from 20 controls (Bote et al., 2012). Pain is a central symptom in FM, and MCP-1 is involved in pain processing and pain sensitivity in the central nervous system (Rodriguez-Pinto et al., 2014) and MCP-1 is reported to enhance excitability of nociceptive neurons (Sun et al., 2006). In a study by Bote et al. (2012) MCP-1 was increased along with the pro-inflammatory marker CRP in 25 patients with FM. In line with this, we have previously described increased hsCRP in the same patient sample population (Groven et al., 2019).

The finding of increased levels of MCP-1 in CFS patients in our study is in contrast to Wyller et al. (2015) not finding any difference in MCP-1 when comparing adolescent CFS patients to controls. However, adolescent patients with CFS may be different from the adult population we examined and to our knowledge there are few studies on MCP-1 in CFS, the field needs further exploration.

Monocyte/macrophage production of MCP-1 and hepatocyte CRP production are both stimulated by the same pro-inflammatory cytokines IFN-γ, IL-1β, IL-6 and TNF-α. As both CRP and MCP-1 are increased in patients in our study, one might expect IFN-γ, IL-1β, IL-6 and TNF-α to be increased too. However, they were not, and the explanation for this is not obvious.

### 4.2 Other immune markers

IFN-γ was lower in FM than for CFS and controls, CFS and controls no differing significantly. In line with this, most studies done on CFS patients found no differences between patients and control groups for IFN-γ (Blundell et al., 2015). Contradicting our findings, an increase of IFN-γ has been reported in FM patients (Behm et al., 2012).

CFS and FM patients had lower levels compared to controls for pro-inflammatory IL-1β, IL-6 and TNF-α. Some studies support our findings of lower plasma IL-1β in FM patients (Ernberg et al., 2018), lower plasma IL-6 levels in CFS patients (Horrnig et al., 2015) and FM patients (Ernberg et al., 2018), and decreased serum TNF-α in FM patients (Hernandez et al., 2010). However, conflicting reports exist for IL-1β, IL-6 and TNF-α in both FM patients (Uceyler et al., 2011) and CFS patients (Blundell et al., 2015; Lyall et al., 2003). Also, we found a tendency towards increased plasma TNF-α in CFS patients (Groven et al., 2018).

Anti-inflammatory IL-10 levels were reduced in FM patients compared to healthy controls in our study. This also has been reported by others (Behm et al., 2012).

Cytokines often regarded as regulatory (IL-17A, IL-4, TGF-β1, TGF-β2 and TGF-β3) were lower in both CFS and FM patients compared to the healthy control group in our study. Interestingly, in the CFS group, only CFS patients with short duration of illness differed significantly from controls in these cytokines. To our knowledge studies on this are scarce and results are conflicting (Blundell et al., 2015; Uceyler and Hauser, 2011).

We hypothesized an increased inflammatory state in CFS and FM patients compared to controls. We have reported increased pro-inflammatory MCP-1 but the four pro-inflammatory cytokines IFN-γ, IL-1β, IL-6 and TNF-α were significantly reduced in our patient population, and not higher, as might be expected given the increased CRP and MCP-1. This may appear a paradox. However, it indicates that the mechanisms are complex and may be related to other factors than diagnoses alone and that these molecules are indirectly or secondarily associated to the pathogenesis of the disorder. Also, these cytokines are not mutually exclusive, for example will upregulated production of MCP-1 by IFN-γ also increase the production of IL-4 (Murphy, 2008). We also found reduced levels in CFS and FM compared to controls for the anti-inflammatory cytokine IL-10, which is in line with a pro-inflammatory state in these disorders. The lower levels of regulatory cytokines (IL-17A, IL-4, TGF-β1, TGF-β2 and TGF-β3) found in plasma of patients with FM and short duration of illness in CFS, could suggest that this regulatory influence is diminished, leading to further imbalances in the immune system of these patients. However, subscribing a strict role of these cytokines in CFS and FM should be taken cautiously, and more studies are needed.

We attempted to discriminate CFS from FM to explores similarities and differences in immune activity in the two groups. There is an overlap between FM and CFS. Only five FM patients (9%) could be considered fibromyalgia cases without fatigue. Yet, the screening of FM patients as part of the diagnostic evaluation in the clinic concluded that these patients did not fulfil the Fukuda et al., (1994) CFS criteria for diagnosis. Similarly, 28% of the CFS patients in our study also were diagnosed with fibromyalgia according to the ACR (1990) criteria.

Sub-dividing the patient group further and comparing the groups pure FM (n ​= ​58), CFS without FM (n ​= ​36) and those with a combination of FM and CFS (n ​= ​13), did not show any difference between the three groups for any of the cytokines apart from IFN-γ. For IFN-γ the patients with FM in either group differed from patients without FM. This supports our finding that IFN-γ is different for FM symptomology.

The main aim and finding in the present study was to compare suffering patients from healthy controls. The field is constantly being explored and some features of CFS and FM are not strictly distinct, and both categories may well be heterogeneous and include other related disorders with unknown aetiology. Thus, the findings still are interesting.

### 4.3 Confounding factors

Several potential confounding factors were tested. Increased MCP-1 was correlated with increased age, BMI, and score on HADS depression scale in subgroups as well as total population and decreased activity in CFS. Levels of regulatory cytokines like TGF and IL-4 were negatively associated with HADS depression score and with short duration of illness in CFS. Increased IP-10 was seen after menopause. No associations were seen between levels of cytokines and fatigue, pain, smoking, type and use of medication, use of birth control and stage of menstrual cycle.

Age and BMI could influence the results, as both high age and BMI are associated with higher inflammation (Poledne et al., 2009; Rea et al., 2018). In our study the CFS group was younger than both FM and controls; and CFS patients and controls had lower BMI than the FM group. This pattern does not fit with age and BMI being responsible for group differences. Also, the patient groups with high MCP-1 had lower levels of other proinflammatory cytokines making the hypotheses general inflammation less likely.

MCP-1 might be related to degree of severity of disorder, for which activity may be an indicator. In line with this the most inactive CFS patients (only move/walk to conduct core tasks) also had the highest levels of MCP-1 compared to the active patients. The control group still having significantly lower levels of MCP-1 than both active and inactive patients (data not shown). Maes et al. (2012) emphasize that there is a chronic fatigue spectrum, suggesting a model with three categories with a continuum of increasing severity of illness. This indicates that the severity of illness should be taken into account in studies on CFS and related conditions.

Like others (Hornig et al., 2015) we found certain differences in expression of cytokines in CFS patients with short and long duration of CFS. CFS patients with an illness duration less than three years had significantly lower levels of TNF-α and IL-10 in our study, while CFS lasting more than 3 years could not be distinguished from controls regarding these cytokines. This difference in short and long lasting disorder is described by others (Broderick et al., 2010; Hornig et al., 2015). Thus, duration of illness and treatment should be taken into account when looking at immune deviations for these disorders.

### 4.4 Limitations

The lack of validated standard measures for cytokines and chemokines is a constant challenge in studies like ours. However, though kits of reagents vary in sensitivity, the relative concentrations/patterns between individuals tested in the same kit is valid. All immune marker samples in this study were analysed by one experienced person in the same lab, using the same assay, and run at the same time. Hence, the cytokine and chemokine pattern for each sample should not be affected.

The groups were not age-matched, and associations between immune markers and age were found. Due to the distribution of our data (samples below the detection limits), the possible confounding factors influencing immune marker levels could not be controlled for, and were only reported as significant associations, thus leading to possible bias in our results.

The diagnostic groups FM and CFS are purely clinically based and with no objective paraclinical measures, and the recruitment of patients from a university specialist clinic only may not be representative for a patient population cohort. However, the patients were referred to this clinic from general practitioners in the primary health care, and we have used strict diagnostic criteria by a specially trained group of specialists in a specialised chronic fatigue and pain centre and this reduces this possible limitation as much as possible. The control group consisted of mainly hospital and university staff, which may not in all aspects represent the general population.

The size of the groups might be larger – especially in the field of deviating findings. However, our groups are large compared to most other studies with these strict inclusion criteria and were based on power calculations.

Another objection is the selection of cytokines and chemokines. It was based on previous reports, available tests and an attempt to cover a broad array of “immune arms”. The role of inflammation and cytokines/chemokines in the immune system as well as other systems like nerve systems is not at all fully understood, thus the relevance of all markers is not absolute. However, this goes for all studies in this field at the moment.

Our study, based on 107 patient samples and 53 healthy control test samples, is a relatively large study in comparison to similar research in this field. The size of the groups, the strict diagnostic criteria and the high competency at the lab where all samples are run at the same time with the same equipment and the same person are all strengths of the study, as well as the competency of the group in the clinic and laboratory. Also, the Caucasian population in this study is rather homogenous with a rather good and homogenous health, status of living etc.

### 4.5 Further research

Clinical overlap of the two diagnoses CFS and FM is well established (Clauw, 2010) and both diagnostic groups probably are heterogenous. Our findings support the hypothesis that CFS and FM share some overlapping immunological similarities. The inconclusive findings in other studies is in line with this. However, so far it cannot be ruled out that immune activity is related to aetiology or pathogenesis of the conditions. If there is an aetiological or pathological function of immune markers in CFS and FM prevention and treatment (*e.g*. blocking MCP-1 activity) could be beneficial to these patients. Substances blocking MCP-1 are under testing for treatment of neuroinflammation, cardiovascular inflammation (Franca et al., 2017), inflammatory disorders (Zoja et al., 2015) and might thus even be of interest in FM and CFS if our findings of increased MCP-1 are confirmed and found to have a function in pathogeneses. Thus, the field should be further explored.

## 5 Conclusion

There were increased levels of MCP-1 in both patient groups, *i.e.* findings in line with previously reported increase in hsCRP in our study population. However, it was unexpected that several other immune markers measured were significantly lower for the same patients. The CFS and FM patient groups were significantly lower than controls in plasma levels for IL-1β, IL-4, IL-6, TNF-α, TGF-β1, TGF-β2, TGF-β3, IL-10 and IL-17A. However, solely based on plasma samples for these immune markers, there were no differences between the CFS and FM patient groups, all together supporting the assumption that these two disorders show overlapping features.

## Funding

This research did not receive any specific grant from funding agencies in the public, commercial, or not-for-profit sectors. The first author has received a PhD grant from the Central Norway Region Health Authority for carrying out the study. This funding source had no involvement in the study design; in the collection, analysis and interpretation of data; in the writing of the report; in the preparation of the article; nor in the decision to submit the article for publication.

## Declaration of competing interest

All the authors declare no conflict of interest.

## Footnotes

1 Sub-groups analyses for age, BMI, HADS, FDSC, fatigue, pain and activity level are found in supplementary Table 1.
