# Supplementary material for: MCP-1 is increased in patients with CFS and FM, whilst several other immune markers are significantly lower than healthy controls
Source: Brain Behav Immun Health. 2020 Mar 28;4:100067. doi: 10.1016/j.bbih.2020.100067 (PMC8474618; doi:10.1016/j.bbih.2020.100067)
Supplement: Multimedia component 1 [file mmc1.docx]

| Supplementary Table 1 | | | | | | | | | | | | | | | | | | | | | |
| --- | --- | --- | --- | --- | --- | --- | --- | --- | --- | --- | --- | --- | --- | --- | --- | --- | --- | --- | --- | --- | --- |
| *Correlation analysis between immune markers and confounding factors in the sub-groups Chronic Fatigue Syndrome (CFS), fibromyalgia (FM) and healthy contols* | | | | | | | | | | | | | | | | | | | | | |
|  |  |  |  |  |  |  |  |  |  |  |  |  |  |  |  |  |  |  |  |  |  |
|  |  | Age | |  | BMI | |  | HADS-D | |  | Fatigue | |  | Pain | |  | FS | |  | Activity | |
|  |  | ρ | *Sign.* |  | ρ | *Sign.* |  | ρ | *Sign.* |  | ρ | *Sign.* |  | ρ | *Sign.* |  | ρ | *Sign.* |  | ρ | *Sign.* |
|  |  |  |  |  |  |  |  |  |  |  |  |  |  |  |  |  |  |  |  |  |  |
| CFS | MCP-1 |  |  |  | .337 | .022 |  |  |  |  |  |  |  |  |  |  |  |  |  | -.359 | .012 |
|  | IP-10 |  |  |  |  |  |  | .287 | .048 |  |  |  |  |  |  |  |  |  |  |  |  |
| FM | MCP-1 | .423 | .001 |  | .364 | .005 |  |  |  |  | .290 | .021 |  |  |  |  |  |  |  |  |  |
|  | IP-10 |  |  |  |  |  |  |  |  |  |  |  |  |  |  |  |  |  |  |  |  |
|  | IFN-γ | -.328 | .012 |  |  |  |  |  |  |  |  |  |  |  |  |  |  |  |  |  |  |
|  | IL-4 |  |  |  | .270 | .042 |  |  |  |  |  |  |  |  |  |  |  |  |  |  |  |
|  | IL-6 |  |  |  | .282 | .034 |  |  |  |  |  |  |  |  |  |  |  |  |  |  |  |
|  | TGF-β1 |  |  |  |  |  |  |  |  |  |  |  |  |  |  |  | .340 | .009 |  |  |  |
|  | TGF-β2 |  |  |  |  |  |  |  |  |  |  |  |  |  |  |  | .294 | .024 |  |  |  |
|  | TGF-β3 |  |  |  | .284 | .032 |  |  |  |  |  |  |  |  |  |  | .320 | 0.14 |  |  |  |
| Controls | MCP-1 | .427 | .003 |  | .428 | .001 |  |  |  |  |  |  |  |  |  |  |  |  |  |  |  |
|  | IFN-γ |  |  |  | .345 | .011 |  |  |  |  |  |  |  |  |  |  |  |  |  |  |  |
|  |  |  |  |  |  |  |  |  |  |  |  |  |  |  |  |  |  |  |  |  |  |
| Note: BMI = body mass index. HADS-D = Hospital Anxiety and Depression Scale, depression score. FS = Fibromyalgia Severity score. Fatigue = Chalder Fatigue Scale. Pain = Numeric rating scale of perceived pain. Only immune markers that were significantly correlated to any of the confounding factors are listed for each sub-group. | | | | | | | | | | | | | | | | | | | | | |
| Correlation analysis: Spearman's rho (ρ). Significance levels were set to *p* < .05 | | | | | | | | | | | | | | | | | | | | | |
|  |  |  |  |  |  |  |  |  |  |  |  |  |  |  |  |  |  |  |  |  |  |
